# Cation-surface RADA16-based self-assembling peptide hydrogel with high adhesion and osteogenesis promoting bone regeneration

**DOI:** 10.1093/rb/rbag162

**Published:** 2026-08-04

**Authors:** Jiangling Zhou, Jing Gou, Qiandong Yang, Sihao He, Juan Cai, Qiankun Yang, Bo Yu, Qijie Dai, Jiulin Tan, Yingtao Gui, Yuan Guo, Junchao Xing, Tianyong Hou

**Affiliations:** Department of Orthopaedics, Southwest Hospital, Army Medical University (Third Military Medical University), Chongqing 400038, China; Chongqing Engineering Research Center for Advanced Material Design and Intelligent Manufacturing in Bone Repair and Reconstruction, Department of Orthopaedics, Southwest Hospital, Army Medical University (Third Military Medical University), Chongqing 400038, China; National & Regional United Engineering Lab of Tissue Engineering, Department of Orthopaedics, Southwest Hospital, Army Medical University (Third Military Medical University), Chongqing 400038, China; Department of Orthopaedics, Southwest Hospital, Army Medical University (Third Military Medical University), Chongqing 400038, China; National & Regional United Engineering Lab of Tissue Engineering, Department of Orthopaedics, Southwest Hospital, Army Medical University (Third Military Medical University), Chongqing 400038, China; Department of Human Anatomy and Histology‑Embryology, Basic Medical Division, Sichuan College of Traditional Chinese Medicine, Mianyang 610065, China; Department of Orthopaedics, Southwest Hospital, Army Medical University (Third Military Medical University), Chongqing 400038, China; Chongqing Engineering Research Center for Advanced Material Design and Intelligent Manufacturing in Bone Repair and Reconstruction, Department of Orthopaedics, Southwest Hospital, Army Medical University (Third Military Medical University), Chongqing 400038, China; National & Regional United Engineering Lab of Tissue Engineering, Department of Orthopaedics, Southwest Hospital, Army Medical University (Third Military Medical University), Chongqing 400038, China; Department of Orthopaedics, Southwest Hospital, Army Medical University (Third Military Medical University), Chongqing 400038, China; Chongqing Engineering Research Center for Advanced Material Design and Intelligent Manufacturing in Bone Repair and Reconstruction, Department of Orthopaedics, Southwest Hospital, Army Medical University (Third Military Medical University), Chongqing 400038, China; National & Regional United Engineering Lab of Tissue Engineering, Department of Orthopaedics, Southwest Hospital, Army Medical University (Third Military Medical University), Chongqing 400038, China; Department of Orthopaedics, Southwest Hospital, Army Medical University (Third Military Medical University), Chongqing 400038, China; Chongqing Engineering Research Center for Advanced Material Design and Intelligent Manufacturing in Bone Repair and Reconstruction, Department of Orthopaedics, Southwest Hospital, Army Medical University (Third Military Medical University), Chongqing 400038, China; National & Regional United Engineering Lab of Tissue Engineering, Department of Orthopaedics, Southwest Hospital, Army Medical University (Third Military Medical University), Chongqing 400038, China; Department of Orthopaedics, Southwest Hospital, Army Medical University (Third Military Medical University), Chongqing 400038, China; Chongqing Engineering Research Center for Advanced Material Design and Intelligent Manufacturing in Bone Repair and Reconstruction, Department of Orthopaedics, Southwest Hospital, Army Medical University (Third Military Medical University), Chongqing 400038, China; National & Regional United Engineering Lab of Tissue Engineering, Department of Orthopaedics, Southwest Hospital, Army Medical University (Third Military Medical University), Chongqing 400038, China; Department of Orthopaedics, Southwest Hospital, Army Medical University (Third Military Medical University), Chongqing 400038, China; Chongqing Engineering Research Center for Advanced Material Design and Intelligent Manufacturing in Bone Repair and Reconstruction, Department of Orthopaedics, Southwest Hospital, Army Medical University (Third Military Medical University), Chongqing 400038, China; National & Regional United Engineering Lab of Tissue Engineering, Department of Orthopaedics, Southwest Hospital, Army Medical University (Third Military Medical University), Chongqing 400038, China; Department of Orthopaedics, Southwest Hospital, Army Medical University (Third Military Medical University), Chongqing 400038, China; Chongqing Engineering Research Center for Advanced Material Design and Intelligent Manufacturing in Bone Repair and Reconstruction, Department of Orthopaedics, Southwest Hospital, Army Medical University (Third Military Medical University), Chongqing 400038, China; National & Regional United Engineering Lab of Tissue Engineering, Department of Orthopaedics, Southwest Hospital, Army Medical University (Third Military Medical University), Chongqing 400038, China; Department of Orthopaedics, Southwest Hospital, Army Medical University (Third Military Medical University), Chongqing 400038, China; Chongqing Engineering Research Center for Advanced Material Design and Intelligent Manufacturing in Bone Repair and Reconstruction, Department of Orthopaedics, Southwest Hospital, Army Medical University (Third Military Medical University), Chongqing 400038, China; National & Regional United Engineering Lab of Tissue Engineering, Department of Orthopaedics, Southwest Hospital, Army Medical University (Third Military Medical University), Chongqing 400038, China; Department of Orthopaedics, Southwest Hospital, Army Medical University (Third Military Medical University), Chongqing 400038, China; Chongqing Engineering Research Center for Advanced Material Design and Intelligent Manufacturing in Bone Repair and Reconstruction, Department of Orthopaedics, Southwest Hospital, Army Medical University (Third Military Medical University), Chongqing 400038, China; National & Regional United Engineering Lab of Tissue Engineering, Department of Orthopaedics, Southwest Hospital, Army Medical University (Third Military Medical University), Chongqing 400038, China; Department of Orthopaedics, 958th Hospital of Chinese People’s Liberation Army (Third Military Medical University), Chongqing 400038, China; Department of Orthopaedics, Southwest Hospital, Army Medical University (Third Military Medical University), Chongqing 400038, China; Chongqing Engineering Research Center for Advanced Material Design and Intelligent Manufacturing in Bone Repair and Reconstruction, Department of Orthopaedics, Southwest Hospital, Army Medical University (Third Military Medical University), Chongqing 400038, China; National & Regional United Engineering Lab of Tissue Engineering, Department of Orthopaedics, Southwest Hospital, Army Medical University (Third Military Medical University), Chongqing 400038, China; Department of Orthopaedics, Southwest Hospital, Army Medical University (Third Military Medical University), Chongqing 400038, China; Chongqing Engineering Research Center for Advanced Material Design and Intelligent Manufacturing in Bone Repair and Reconstruction, Department of Orthopaedics, Southwest Hospital, Army Medical University (Third Military Medical University), Chongqing 400038, China; National & Regional United Engineering Lab of Tissue Engineering, Department of Orthopaedics, Southwest Hospital, Army Medical University (Third Military Medical University), Chongqing 400038, China

**Keywords:** positive charge surface modification, self-assembling peptide hydrogel, TRSAW, cell adhesion, osteogenesis

## Abstract

Despite the widespread use of various bone repair materials, ideal scaffolds with excellent cell adhesion and satisfactory osteogenesis are still urgently needed for bone defect regeneration. The self-assembling peptide RADA16‑I hydrogel has been widely used for bone defect repair owing to its excellent biocompatibility and favorable cytocompatibility, yet its application is limited by insufficient osteogenesis and weak cell adhesion. In this study, a novel peptide RADA16‑TRSAW was designed by conjugating the positively charged osteogenic short peptide TRSAW to RADA16‑I via solid-phase synthesis. TRSAWmax (mixture of RADA16‑TRSAW and RADA16‑I as 1:1) carried a net positive charge, maintained a stable β‑sheet secondary structure, retained self-assembly capability, and readily formed a hydrogel in PBS. The resulting hydrogel showed a microstructure and rheological properties similar to those of RADA16‑I hydrogel, suitable for cell growth, but with significantly enhanced cell adhesion owing to cationic arginine residues. RNA‑seq confirmed the upregulation of adhesion-related genes and pathways. Moreover, TRSAWmax exhibited excellent biocompatibility, promoted hMSC osteogenic differentiation through the Src‑Erk pathway *in vitro*, and demonstrated superior osteogenic capability *in vivo*. This work provides a highly adhesive and osteogenic peptide hydrogel candidate for accelerating bone tissue regeneration.

## Graphical abstract

**Graphical Abstract:**
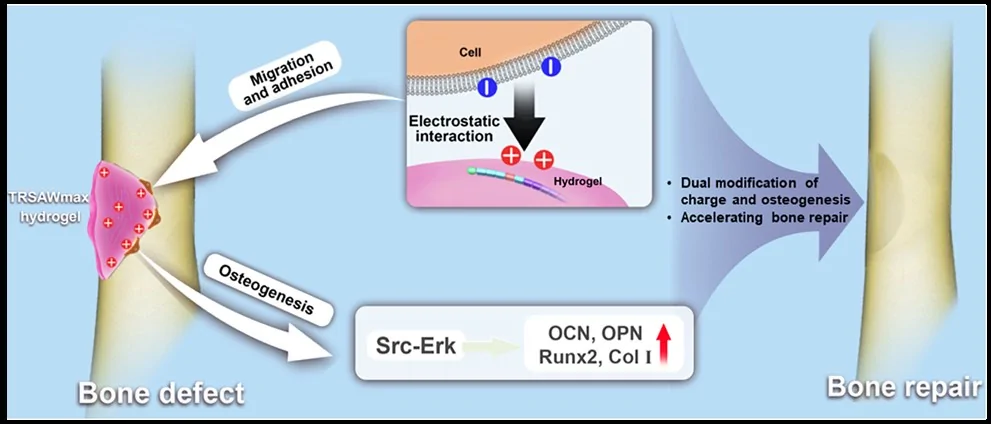

## Introduction

Repairing bone defects caused by trauma, tumor resection, infection or osteoporosis remains a major clinical challenge in the clinic [1, 2]. Despite the availability of autologous bone grafts, allografts and numerous synthetic bone repair materials, clinical outcomes are often limited by inadequate integration, poor biocompatibility, inflammatory risks or lack of bioactivity to effectively support bone regeneration [3–5]. Ideal scaffolds for bone tissue engineering are urgently needed to offer favorable biocompatibility, excellent bioactivity and biodegradability, along with simple synthesis and low cost for efficient bone reconstruction [6, 7]. In recent years, a wide range of advanced biomaterial platforms have also been successfully developed to effectively enhance osteogenic activity and promote bone regeneration [8–12]. Despite these advances, the translatable scaffolds in the clinic that simultaneously achieve strong cell adhesion, stable self-assembly and potent osteogenesis without exogenous growth factors or complex components remain scarce.

Among various biomaterials, self‑assembling peptide hydrogels have emerged as highly promising 3D scaffolds owing to their excellent biocompatibility, tunable biodegradability, ECM‑like nanofibrous architecture and accurate molecular designability. Extensive research has established a rich library of self‑assembling peptide systems with distinct structural and functional characteristics [13–15]. The numerous studies have confirmed that rationally designed ionic self‑complementary peptides, represented by RADA16‑I, can spontaneously undergo hierarchical self‑assembly into stable β‑sheet nanofiber networks under physiological conditions, forming highly hydrated hydrogels with favorable cytocompatibility and controlled degradability. Owing to these merits, RADA16‑I has been extensively explored as a versatile scaffold for the regeneration of bone, cartilage, neural and skin tissues [16, 17]. However, the use of pure RADA16-I for bone defect repair lacks sufficient osteogenesis and proactively captures cells. Fortunately, it has been shown that RADA16-I can be readily modified by directly adding functional amino acid sequences to the amino or carboxyl terminus during solid-phase synthesis, and appropriate adjustment of the ratio does not affect the secondary structure of RADA16-I but confers additional functions [18–22]. Quite a few bioactive peptides, such as IKVAV, RGIDKRHWNSQ, EVYVVAENQQGKSKA, KLTWQELYQLKYKGI and PRGDSGYRGDS were successfully added to RADA16-I to promote cell proliferation, migration, adhesion and vascularization [23]. To achieve more functions or enhance certain properties, researchers co-assembled two or more modified RADA16-I peptides. However, this strategy is costly, time-consuming and more difficult to fabricate into a hydrogel. Therefore, creating a polypeptide with both high adhesiveness and osteogenic performance based on RADA16-I for bone repair is highly necessary.

TRSAW (Thr-Arg-Ser-Ala-Trp), also known as osteostatin, is a pentapeptide derived from the C-terminal domain of parathyroid hormone (PTH). PTH is an 84-amino acid polypeptide secreted by the parathyroid glands under the control of serum calcium and phosphate levels [24, 25]. It primarily acts on osteoid to enhance calcium reabsorption and calcium release. Several bioactive epitopes have been identified in PTH, including parathyroid hormone-related protein (PTHrP) (1–34), PTHrP (1–36), PTHrP (107–139) and osteostatin (107–111). Through its N- and C-terminal domains, parathyroid hormone-related peptide (PTHrP) can promote the reversal of bone loss in animal models of osteoporosis by stimulating osteoblastic function [26–28]. It is worth noting that among these epitopes, PTHrP 107–111(TRSAW) demonstrates excellent osteogenic properties *in vitro* and the ability to stimulate bone accrual *in vivo*. When implanted into bone defects, TRSAW provides substantial benefits for osteoprogenitor proliferation, connective tissue formation and revascularization [29–31]. These findings suggest that TRSAW could be a promising target for designing biomaterials in the field of bone tissue engineering. At the same time, TRSAW carries a net positive charge derived from the arginine residue (R), and based on the interaction between positive and negative charges, it has the potential to enhance cell adhesion and capture through electrostatic action with negatively charged cells.

Based on the abovementioned statement, we innovatively modified RADA16-I with the positively charged functional short peptide TRSAW in this study to endow it with stronger cell adhesion and improved osteogenic capacity for bone repair. First, a RADA16-TRSAW composite was successfully developed by conjugating the small functional peptide TRSAW to the C-terminus of RADA16-I via solid-phase synthesis. A mixture of RADA16-I and RADA16-TRSAW solutions at a 1:1 volume ratio was designated as TRSAWmax, and the secondary structure and microstructure of the peptide solutions were observed. It was surprising to find that TRSAWmax maintained a high proportion of β-sheet structure and self-assembly performance, and could be easily fabricated into a hydrogel induced by salt ions. The physicochemical properties of the hydrogels, including pore structure, rheological properties and stability, were then investigated. Moreover, it was evaluated whether the positive charge attached to the short peptide interacted with the negative charge carried by cells to promote cell adhesion and facilitate cell growth. Furthermore, the adhesion-related genes and pathways in hBMSCs activated by TRSAWmax hydrogel were explored through transcriptome sequencing. Meanwhile, the modification of functional short peptides was observed to further enhance the osteogenic differentiation ability of hMSCs *in vitro* and the bone reconstitution ability *in vivo*. Finally, the potential signaling pathway by which the TRSAWmax hydrogel promotes bone defect healing was explored through cell experiments *in vitro*.

## Materials and methods

### Materials

Dulbecco’s modified Eagle’s medium (DMEM)/Hamm’s nutrient mixture F12 (DMEM/F12, 1:1) and fetal bovine serum (FBS) were purchased from Wuhan Procell Biotechnology Co., Ltd. Osteogenic differentiation medium (HUXMX-90021) was obtained from Cell Cyagen Bioscience, China. Trypsin-EDTA (0.25%) was supplied by Hyclone, Logan, UT. The Cell Counting Kit-8 (CCK-8, CK04), phosphate buffer saline (PBS, P1000-100PCS), EDTA decalcifying solution (pH 7.2, E1171-500 mL) and 4% paraformaldehyde (P1110-500 ml) were all obtained from Solarbio Life Sciences. The fluorogenic ester Calcein-AM (CAM) was purchased from Dojindo and the Prime Script^TM^ RT reagent Kit with gDNA Eraser was obtained from TaKaRa. TRIzol® reagent and SuperScript® III Reverse Transcriptase were supplied by Invitrogen. DAPI solution (C1005), protein extraction kit (P0028), chemiluminescent agents (P0018S), Alkaline Phosphatase (ALP) Assay Kit (P0321S) and Alizarin Red S (ARS) staining kit were all purchased from Beyotime Biotechnology Co., Ltd. (Jiangsu, China). Src inhibitor PP1 and Erk1/2 inhibitor SCH772984 were acquired from Selleck Chemical, USA, and MedChem Express, USA, respectively. The primary antibodies against OCN (DF12303), RUNX2 (AF5186), COL-Ι (AF7001) were obtained from Affinity Biosciences; Src (2109S) and Erk1/2 (4695T), phosphorylation of Src and Erk1/2 (59548S and 4377T) were obtained from Cell Signaling Technology, Inc. (Danvers, MA, USA); OPN (BM4208) was supplied by BOSTER; VEGF (19003-1-AP) was obtained from Proteintech. Antibody against GAPDH (BTL1029) and secondary antibody (BSA1013) were supplied by Biospes Co., Ltd. (Chongqing, China). The primers used for reverse transcription polymerase chain reaction (RT-PCR) were synthesized by Sangon Biotech Co., Ltd. (Shanghai, China). The peptides (RADA16-I, RADA16-TRSAW and RADA16-TASAW) were provided by QYAOBIO (ChinaPeptides Co., Ltd.). Chemical reagents such as *N*,*N*-dimethylformamide (DMF, 73.09 g/mol), dichloromethane (DCM, 84.93 g/mol), diisopropylethylamine (DIEA, 129.25 g/mol), hexafluorophosphate benzotriazole tetramethyl yronium (HBTU, 179.20 g/mol), trifluoroacetic acid (TFA, 114.02 g/mol), 1,2-ethanedithiol (EDT, 94.2 g/mol) and triisopropylsilane (TIS, 118.28 g/mol) were sponsored by QYAOBIO (ChinaPeptides Co., Ltd.) with purity higher than 90%.

Human mesenchymal stem cells from bone marrow (hBMSCs) were purchased from Cell Cyagen Bioscience, China (HUXMA-01001). Male C57BL/6J mice (6–8 weeks old) were obtained from HFK Bioscience Co., Ltd. (Beijing, China).

### Peptide synthesis and hydrogel preparation

The peptides used in this study, RADA16-I (Ac-RADARADARADARADA-CONH2), RADA16-TRSAW (Ac-RADARADARADARADAGGTRSAW-CONH2) and RADA16-TASAW (Ac-RADARADARADARADAGGTASAW-CONH2), were synthesized by solid-phase peptide synthesis with the assistance of a 12-channel semi-automatic peptide synthesizer. All peptides were elongated from the C-terminal to the N-terminal, and RADA16-TRSAW or RADA16-TASAW was obtained by coupling the pentapeptide TRSAW or TASAW to the C-terminus of RADA16-I by a universal linker (GG), as illustrated in Figure 1A and B. Briefly, 2-Chlorotrityl Chloride Resin (degree of substitution: 1.03 mmol/g) and appropriate volume of DMF (15 mL/g) were put into the reaction tube and oscillated for 30 min. Then, the first amino acid was grafted on with the aid of 10 times the molar excess DIEA, and sealed with methanol. After deprotection by 20% piperidine/DMF solution and being washed by DMF (10 mL/g)/DCM (10 mL/g)/DMF (10 mL/g) twice, the remaining amino acids were added sequentially from right to left with the help of 3 and 10 times molar excess of HBTU and DIEA. The obtained polypeptide was cracked by TFA 95%/water 1%/EDT 2%/TIS 2% solution and then precipitated by ether. The pure polypeptide powder was obtained via high-performance liquid chromatography (HPLC) followed by freeze-drying, and was set aside at −20°C for use.

**Figure 1:**
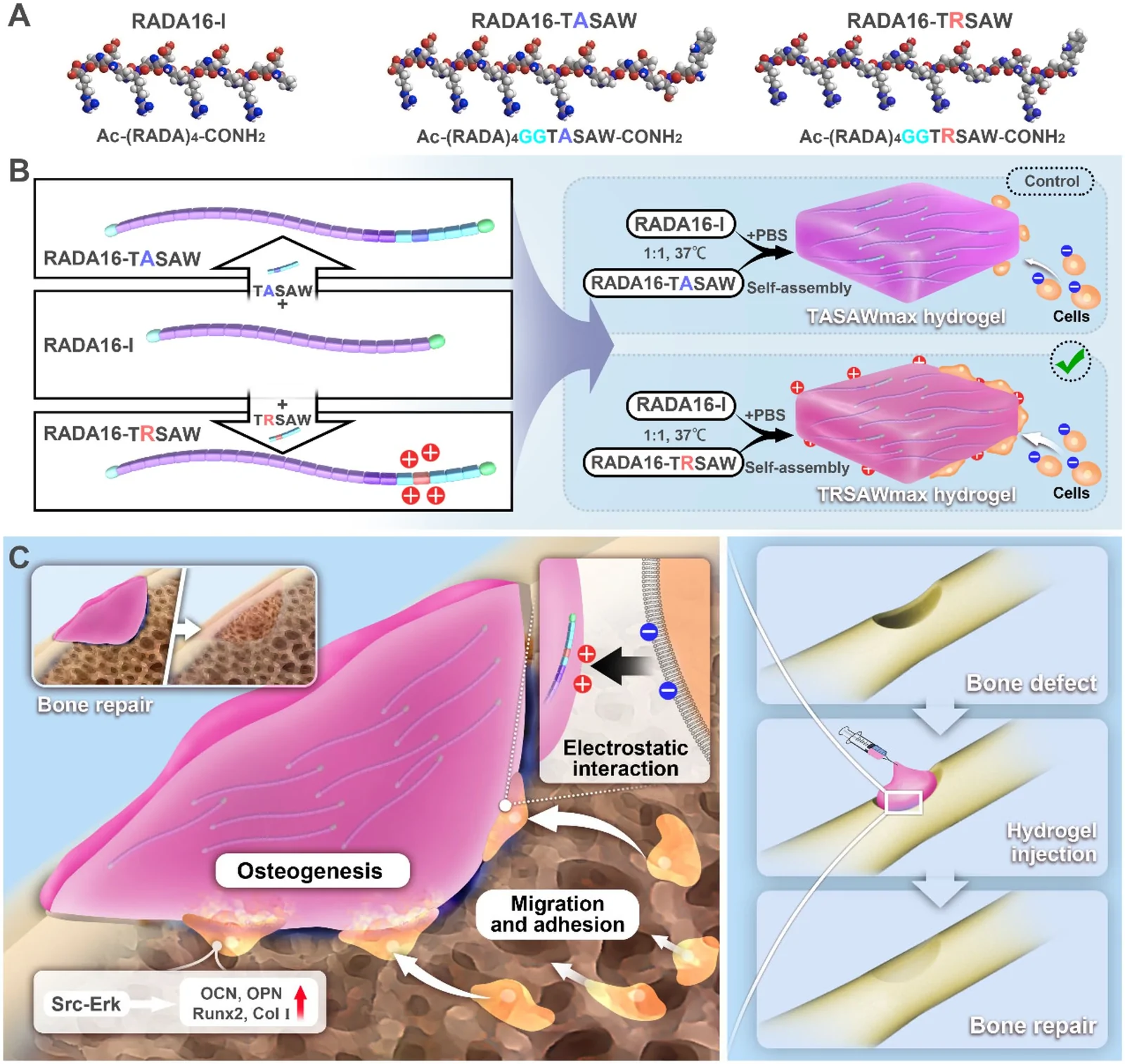
Schematic illustration of the self-assembling peptide hydrogel for bone defect treatment. (**A**) The amino acid sequences and molecular models of all peptides are shown in the extended direction (N→C). Color code: white for hydrogen, cyan for carbon, red for oxygen and blue for nitrogen. (**B**) The self-assembly process of TRSAWmax and TASAWmax hydrogels in PBS solution at 37°C owing to its positive surface charge, the TRSAWmax hydrogel can adhere to cells more effectively. (**C**) Mechanism and process by which injectable TRSAWmax hydrogel promotes bone repair. (TRSAWmax: a mixture of RADA16-I and RADA16-TRSAW solutions mixed at a 1:1 volume ratio; TASAWmax: a mixture of RADA16-I and RADA16-TASAW solutions mixed at a 1:1 volume ratio.).

The N-termini of these peptides were acetylated, and the C-termini were amidated, and the molecular models of each peptide (Figure 1A) were constructed using a free modeling software (ChemBioDraw Ultra 14. 0). The purities and molecular weight of synthetic peptides were tested by HPLC chromatogram and ESI-MS spectrum. The final purities are all more than 90%.

RADA16-I, RADA16-TRSAW and RADA16-TASAW peptide powders were dissolved in Milli-Q water at a concentration of 1% (w/v, 10 mg/mL) as stock solutions with the help of 30 min ultrasound, and the TRSAWmax or TASAWmax peptide stock solution was obtained by mixing the RADA16-I and RADA16-TRSAW or RADA16-TASAW solutions at a volume ratio of 1:1 with sonication for 30 min. All of the 1% peptide solutions were diluted in distilled water to a working concentration of 0.5% (w/v, 5 mg/mL) for the following tests. All of the gelation scaffolds were prepared with equivalent phosphate-buffered saline (pH = 7.4) triggering at 37°C, as shown in Figure 1B, and the formed hydrogels were washed with PBS thrice more to equilibrate the hydrogel to a physiological pH. Macroscopic appearance images of the solution and hydrogel for RADA16-I and TRSAWmax were taken by a digital camera (80D, Canon, China).

### Physicochemical property analyses

The circular dichroism (CD) spectra were used to investigate the secondary structure of the self-assembling peptide solutions. The stock solutions of RADA16-I, RADA16-TRSAW and TRSAWmax were diluted 100 times at 25°C and equilibrated for 30 s, then 500 μL of sample solution was added to a circular dichroism colorimetric cuvette for detection. A J-810 CD spectrometer (J-810 JASCO Corporation, Tokyo, Japan) was used to measure the entire 190–310 nm wavelength range, and the wavelengths ranging from 190 to 260 nm were used for analysis. In this study, OMNIC software was used to smooth pre-process CD spectral data, and the data was uploaded to the BeStSel platform to evaluate the relative content of secondary structure.

The working solutions of RADA16-I, RADA16-TRSAW and TRSAWmax were diluted to a concentration of 0. 005% (w/v) by reverse osmosis H_2_O and sonicated for 30 min. Then 10 μL of each peptide solution was dropped onto a freshly cleaved mica surface for 20 s and rinsed with 100 μL of distilled water three times. Next, the peptide sample was air-dried, and images were acquired immediately by Atomic Force Microscope (AFM, SPI3800N–SPA400, Seiko, JAPAN) in the gaseous phase. The spring constant of the cantilever was 200 N/m, and the knock mode was operated with a silicon scanning probe.

A cryo-scanning electron microscope (Cryo-SEM, FEI Quanta 450 equipped with Quorum PP3000T refrigerated transmission system) was adopted to detect the microstructure of the peptide solution and hydrogel for RADA16-I and TRSAWmax. The freshly prepared sample was placed on a sample table coated with conductive carbon glue, and frozen in liquid nitrogen slush for 30 s to be broken for a cross-section, and then the sample was transferred under vacuum to the sample preparation chamber for sublimation and sprayed with gold through a cryogenic preparation transport system. The obtained cross-section diagram was observed at a temperature of −140°C and the acceleration voltage of 10 kV.

The rheological characteristics of the hydrogels were measured on a rheometer (TA DHR-1, USA) equipped with a 40 mm flat plate. We first executed strain sweeps from 1% to 50% at a frequency of 1 rad/s to confirm the appropriate strain for the frequency sweeps test. Then frequency sweeps were performed to record the storage modulus (*G*′) and loss modulus (*G*″) over the frequency range of 0.1–100 rad/s at a constant strain of 0.5%. These procedures were carried out at a controlled temperature of 25°C and with a set gap of 1000 μm.

The zeta potential detections of the peptide solution and hydrogels were completed with the aid of Zetasizer Nano S (Malvern Instruments Ltd, Worcestershire, UK) at room temperature. Before detection, each peptide solution was diluted to 0.1% in pure water and adjusted to neutral pH 7.4 with the assistance of NaOH, and the formed hydrogels were broken into particles and dispersed into an aqueous solution under the probe sonication.

The degradation behavior *in vitro* of TRSAWmax hydrogel was assessed under natural PBS immersion and proteinase K-triggered enzymatic degradation. Briefly, 200 μL of hydrogel was loaded into pre-weighed 1.5 mL centrifuge tubes to record the initial gel weight. Samples were supplemented with 200 μL sterile PBS (with or without 5 U/mL proteinase K), followed by incubation at 37°C with shaking at 100 rpm. At predetermined time points, supernatant was discarded and residual hydrogel-containing tubes were weighed; fresh corresponding medium was replenished for subsequent incubation.

### Release of the functional self-assembled peptide

The release of the polypeptide was detected based on the feature that aromatic amino acids had a specific absorption peak in the ultraviolet spectrophotometer. About 200 μL of RADA16-TRSAW or TRSAWmax stock solutions was induced into a solidified state by PBS at 37°C for 2 h, then the obtained hydrogel was incubated in 5 mL PBS for various time scales (1, 3, 5, 7, 9, 12, 24, 36, 48 and 72 h) at room temperature. Two milliliters of solution were collected at different time points for UV detection, and another fresh 2 mL PBS was added to make up the difference. The amount of peptide released into the supernatant solutions was measured by UV-vis spectroscopy at *λ*max = 290 nm.

### Cell culture and seeding on self-assembling peptide hydrogel scaffolds

Cell suspension of hBMSCs was resuspended by an equal volume of PBS, and the mixture was centrifuged again at 1500 rpm for 5 min. The supernatant was removed, and 5 mL of MSC culture medium (DMEM/F12 with 15% FBS) was added. Cells were harvested for further expansion at approximately 80–90% confluence. All the cell experiments were approved by the Ethics Review Committee of Southwest Hospital.

The different peptide solutions (40 μL) were added carefully into the wells of 24-well plates. 100 μL of DMEM/F12 was added, and the samples were incubated for 0.5 h at 37°C. Next, wells were washed with fresh DMEM/F12 three times to equilibrate to a physiological pH of 7.4 before cell seeding. Cells were seeded onto peptide hydrogels at a concentration of 1 × 10^4^ cells/cm^2^.

### Cell adhesion assay

Approximately 2 × 10^4^ cells were added into each well of 24-well plates containing RADA16-I peptide hydrogel, TASAWmax hydrogel, TRSAWmax hydrogel or PBS, which served as a control. Adhesion was allowed to proceed for two different time intervals (1 or 3 h). After the time interval had elapsed, the substrates were washed with PBS three times and then stained with DAPI for cell counting. Briefly, cells were washed with PBS, fixed with 4% paraformaldehyde for 15 min, and then incubated with DAPI solution at room temperature for another 15 min in the dark. After being washed thrice with PBS, the cells stuck on the plates were observed using a fluorescence microscope (CFM-300; Nikon, Japan), and the average number of cells was counted using ImageJ software.

### Transcriptome sequencing and data analysis

The hBMSCs were co-cultured with RADA16-I or TRSAWmax hydrogel directly for 3 days. After the collection of RNA, the cDNA library was constructed, and RNA-seq data were detected using the Illumina Novaseq™ 6000 (LC-Bio Technology CO., Ltd., Hangzhou, China) following the vendor’s recommended protocol. Raw data (raw reads) in fastq format were processed through in-house Perl scripts. The differentially expressed mRNAs were selected with fold change > 2 or fold change < 0.5 and *P* values < 0.05 by DESeq2, and then analyzed the related Kyoto Encyclopedia of Genes and Genomes (KEGG) functional enrichment and Gene Ontology (GO) functional clustering of the differentially expressed mRNAs. Finally, representative gene expression of *RELN*, *ITGA2*, *SPP1*, *LAMC2*, *ITGB3*, and *LAMB3* was evaluated via the q-PCR technique.

### Cell viability and proliferation

After culturing for 7 days, hBMSCs in the peptide hydrogels were labeled with the fluorogenic ester Calcein-AM to detect live cells. Briefly, the scaffolds containing cells were incubated with 2 mM CAM for 30 min at room temperature in the dark and rinsed gently with PBS three times. The number of live cells in each group was determined by counting five randomly collected overlapping areas using a fluorescence microscope (CFM-300; Nikon, Japan).

The proliferation of hBMSCs was measured using the Cell Counting Kit-8. Human BMSCs cultured with control medium were used as controls. After 1, 3, 5, 7, 9 and 14 days of culture, CCK-8 was added into each well at a 1:10 ratio and incubated at 37°C in a 5% CO_2_ incubator for 3 h. The absorbance was then measured at an absorption wavelength of 450 nm using a VersaMax microplate reader (Thermo Fisher Scientific).

### Osteogenic differentiation analysis *in vitro*

Osteogenic differentiation was assessed by RT-PCR and western blot. Cells were cultured with osteogenic differentiation medium. For RT-PCR, total cellular RNA was isolated using TRIzol® reagent after 7 or 14 days of culture. Two micrograms of total RNA were reverse transcribed into cDNA using SuperScript® III Reverse Transcriptase. The synthesized cDNAs were subjected to qPCR using the Prime Script^TM^ RT reagent Kit with gDNA Eraser under the following conditions: initial denaturation at 95°C for 10 min, followed by 39 cycles of denaturation at 95°C for 10 s, an annealing phase of 10 sat 60°C, and extension at 72°C for 40 s. The primer sequences were listed in Table S4. The relative levels of expression of each specific osteogenic gene (*ALP*, *OCN*, *OPN* and *Runx2*) were then normalized to the reference gene Glyceraldehyde-3-Phosphate Dehydrogenase (GAPDH) using the 2 − ΔΔCt method.

For western blot, the nuclear and total proteins were extracted using a protein extraction kit according to the manufacturer’s instructions for 24 h in the presence or absence of Src inhibitor PP1 or Erk1/2 inhibitor SCH772984. Equivalent amounts of protein (20 μg) were separated by sodium dodecyl sulfate polyacrylamide gel electrophoresis and transferred onto polyvinylidene fluoride membranes. After blocking for 1 h, the membrane was incubated with specific antibodies against GAPDH, OPN, OCN, RUNX2, VEGF, COL-Ι, Src, and Erk1/2, phosphorylation of Src and Erk1/2 at 4°C. After washing with PBS, the membrane was incubated with secondary antibodies for 1 h at 37°C. Immunoreactive bands were detected using chemiluminescent agents, and their intensities were quantified using Image-Pro Plus 6.0 software (Media Cybernetics, Rockville, MD).

The ALP activity assay for hBMSCs differentiation was performed at 7 and 14 days of culture in osteogenic differentiation medium. Cells were fixed with 4% paraformaldehyde for 15 min. Each well was stained with the Alkaline Phosphatase Assay Kit according to the manufacturer’s protocol.

The ARS staining was performed to visualize the mineral deposition of hBMSCs at 7 and 14 days. Samples were cleaned with PBS buffer without calcium and magnesium (Sigma-Aldrich Chemicals). After fixation in 4% paraformaldehyde, cells were washed three times and incubated in a solution of 2% Alizarin Red S for 15 min. The samples were washed, air dried and the calcium deposition area was analyzed using five randomly selected fields under an optical microscope (Olympus IX71, Japan).

### *In vivo* osteogenic efficacy

This study was approved by the Ethics Committee of the Army Medical University (AMUWEC20232848). For the *in vivo* therapeutic efficacy of the TRSAWmax hydrogel, C57BL/6J mice were randomly divided into three groups (*n* = 6 per group). PBS served as a negative control. The experimental groups included a blank group, RADA16-I hydrogel and TRSAWmax hydrogel. The femurs of mice were exposed to construct a model of a partial femoral defect. The animals were injected with 50 μL of the control or experimental group. The mice were sacrificed by cervical vertebral dislocation after 6 weeks, and their bone tissues were dissected and fixed in a 4% formaldehyde solution for a series of detection.

After being placed in 4% formaldehyde solution for 1 week, the three-dimensional reconstruction of bone with X-ray and micro-CT (SkyScan1272, BRUKER, Germany) was performed. The volume of new bone was derived from the CT images by delineating the areas of the contrast-enhanced regions of interest (ROI) in the X, Y and Z planes. Images were obtained by adjusting the micro-CT data window display to enhance the edge delineation of the new bone mass. The 3D images of the grafts were reconstructed using a middle-frequency kernel. The quantitative measurements of the new bone were used to calculate bone volume per tissue volume (BV/TV), bone mineral density (BMD), trabecular number (Tb. N) and trabecular thickness (Tb. Th).

In the histological assay, bone tissues were fixed with a 4% formaldehyde solution for 48 h at 4°C, and decalcified with 10% EDTA solution. Then, the samples were embedded in paraffin and sectioned at a thickness of 5 μm for staining with H&E, Masson, COL-I or OCN.

### Statistical analysis

All experiments were repeated at least three times. GraphPad software was used for statistical analysis. All numerical results were expressed as means ± SD. Data were compared using a *t*-test (two groups) or two-way analysis of variance (ANOVA) (multiple groups), followed by Tukey’s multiple comparisons *post hoc* test. *P*-values < 0.05 were considered to be significant.

## Results

### Synthesis and characterization of self-assembled peptide solutions and hydrogels

The synthesis of the peptides used in this study was described in the Materials and Methods section, and HPLC and ESI-MS measurements were performed to confirm their successful synthesis. According to the HPLC results, the purities of RADA16-I and RADA16-TRSAW were approximately 93.03% and 93.26%, respectively, as calculated from the peak area (Figure S1, Table S1). By analyzing the *m*/*z* values of the molecular [M + nH] ^n+^ ions produced from peptide fragments in the ESI-MS spectrum (Figure S2), the molecular weights of RADA16-I and RADA16-TRSAW were determined to be approximately 1712.80 and 2428.57 g/mol, respectively, and the calculated molecular weights were consistent with the theoretical masses of those peptide components (RADA16-I 1671.75 g/mol, RADA16-TRSAW 2387.52 g/mol). These results indicated that we had successfully prepared the two peptides.

The strong tendency to form secondary structures is crucial for peptide self-assembly into a hydrogel matrix, and different secondary structures exhibit specific circular dichroism (CD) characteristics. Thus, we used the CD analysis to reveal the secondary structure of the synthetic peptide. As shown in Figure 2A, a typical spectrum for β-sheet structures, with a negative maximum molar residue ellipticity at 216 nm and a positive maximum at 195 nm, was observed for RADA16-I, and the fitting results (Table S2) showed that the relative contents of α-helix, β-sheet, β-turn and random coil were 9.8%, 58.8%, 7.0% and 24.5%, respectively. The high proportion of β-sheet structure indicated that RADA16-I had a relatively stable secondary structure in solution, which is an important factor in its ability to form a gel. However, this typical β-sheet structure was not obvious in the pure RADA16-TRSAW solutions and was instead replaced by a pronounced random structure, appearing as a negative peak at 195 nm (Figure 2A), while the proportion of β-sheet structure was reduced to 25.9% along with an increasing content of random structure (Table S2), indicating that the stable secondary structure was disrupted rather than the flexible and loose structure and its ability to form a gel might be affected. Interestingly, similar structural characteristics were observed for RADA16-I in a mixed solution TRSAWmax, produced by mixing RADA16-TRSAW and RADA16-I solutions at a volume ratio of 1:1, and the β-sheet content returned to 40.2% higher than that of α-helix (12.1%), β-turn (12.4%) and random structure (35.3%) (Figure 2A and Table S2), which might be attributed to the formation of a hydrophobic core between the molecular chains of RADA16-TRSAW and RADA16-I. Those results were consistent with previous reports [23], indicating the potential of TRSAWmax to self-assemble into a gel.

**Figure 2:**
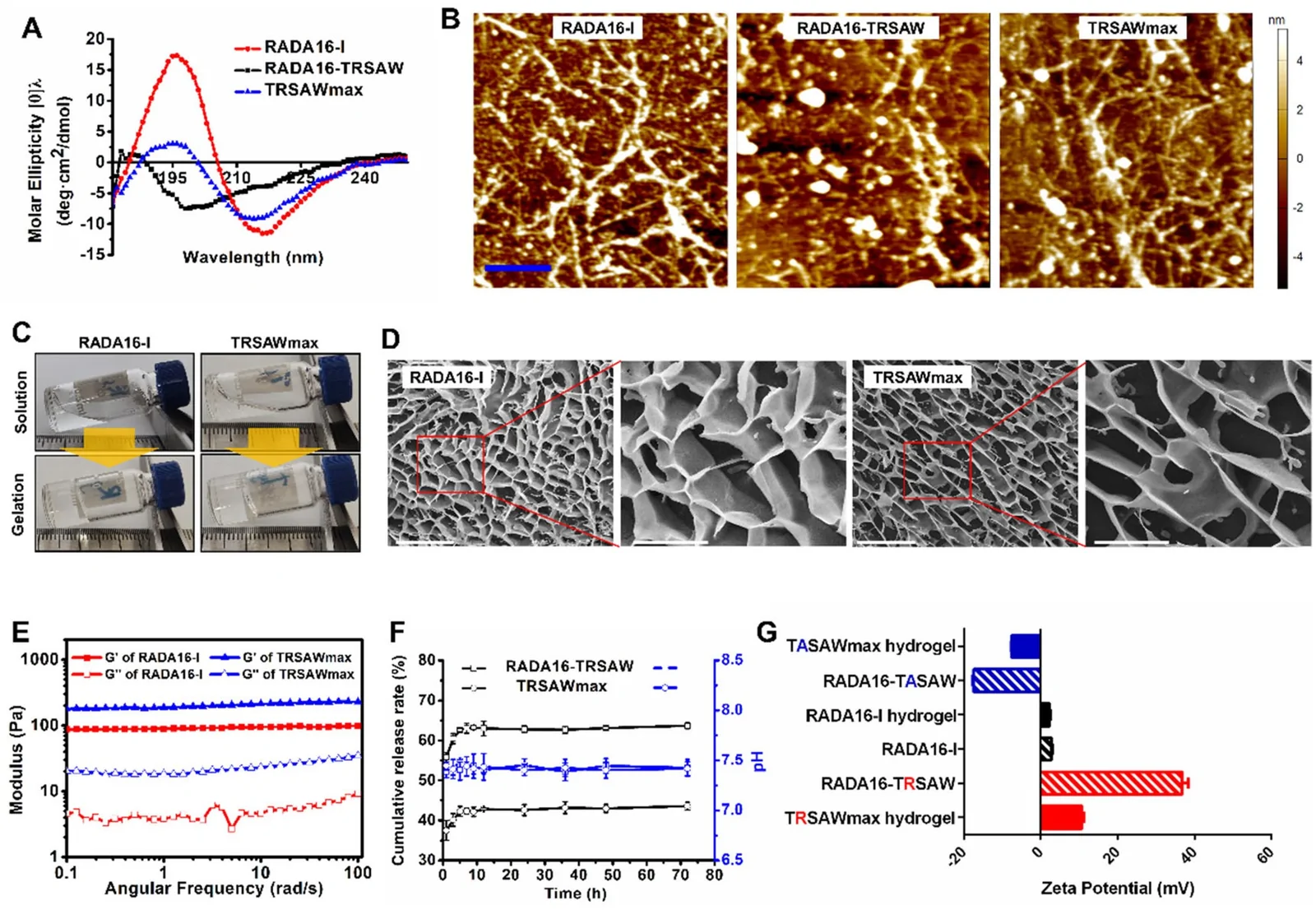
Characterization of peptide solutions and hydrogels. (**A**) Representative circular dichroism (CD) spectra of the peptide solutions (concentration: 0.01% w/v; pH 7.4) at 20°C. (**B**) Atomic force microscopy (AFM) images of three peptides at a 1% concentration. Scale bar: 500 nm. (**C**) Macroscopic images showing the appearance images of the solution and hydrogel for RADA16-I and TRSAWmax. (**D**) Cryo-scanning electron microscopy (Cryo-SEM) image of RADA16-I and TRSAWmax hydrogel. Scale bar: 20 μm. (**E**) Rheological measurements of RADA16-I and TRSAWmax hydrogel across an angular frequency range of 0.1–100 rad/s. (**F**) The pH values (blue) and release profiles (black) of the short peptide TRSAW from two types of self-assembling peptide hydrogels (data points represent the average of three samples). (**G**) Zeta potentials of different peptide solutions and hydrogels (concentration: 0.1% w/v and pH 7.4) at 25°C.

To verify the abovementioned hypothesis, we used atomic force microscopy (AFM) to compare the microscopic structures assembled by different polypeptides. As shown in Figure 2B, all three types of peptides (RADA16-I, RADA16-TRSAW and TRSAWmax) were able to form interwoven nanofibers, indicating their self-assembling properties, although many differences among them still remained. Apparently, sparse interweaves and fewer crosslinks were observed in RADA16-TRSAW than in RADA16-I and TRSAWmax, implying that the self-assembly capacity was greatly affected by the modification of TRSAW but could be restored by mixing with RADA16-I, as supported by the gel-formation assay. Under PBS triggering, both of RADA16-I and TRSAWmax solutions formed a stable hydrogel (Figure 2C), whereas RADA16-TRSAW remained an unstable gel (Figure S3). Therefore, we used TRSAWmax solutions to fabricate a hydrogel for bone repair in this research. The hydrogel precursor solution can be smoothly extruded from a double syringe and rapidly form a stable hydrogel under physiological conditions (Figure S4), directly confirming its excellent injectability and potential for minimally invasive applications.

To observe the microstructure of the hydrogels, we performed cryo-scanning electron microscopy (Cryo-SEM). We found that the formed hydrogels exhibited porous structures, whereas the solutions showed a parallel layered structure (Figure 2D and Figure S5), which was attributed to the crosslinking of salt ions, indicating that gel formation had occurred. In addition, both RADA16-I and TRSAWmax hydrogels exhibited internally interconnected porous network structures. The similar porous structures and sizes reflected that a suitable microstructure for cell growth was maintained by mixing RADA16-I with RADA16-TRSAW. Moreover, the TRSAWmax hydrogel exhibited rheological characteristics similar to those of the RADA16-I hydrogel owing to its analogous microstructure. The rheological graphs showed that the hydrogel’s elastic shear storage modulus (*G*′) was consistently higher than its viscous shear loss modulus (*G*″) as the strain and angular frequency changed (Figure 2E and Figure S6), indicating a solid-like hydrogel state and a relatively stable structure.

The cumulative release assay was used to assess the stability of the hydrogels (Figure 2F). Both TRSAWmax and RADA16-TRSAW hydrogels showed similar release patterns, increasing rapidly at first and then gradually stabilizing, and the release rates of both peptide hydrogels were nearly equal over 10 h. However, the cumulative release of TRSAWmax scaffold was lower than that of the RADA16-TRSAW at each time point. During the entire release cycle, the pH of the hydrogel showed no clear fluctuation and remained within a relatively stable range (Figure 2F, blue line). These results indicated that the TRSAWmax hydrogel was stronger and more stable than RADA16-TRSAW, probably owing to the self-assembly ability of RADA16-I, and may provide a more favorable environment for cell growth. Subsequently, the biodegradation of the TRSAWmax hydrogel was also evaluated through a gel weight loss experiment in the absence or presence of proteinase K (Figure S7). The hydrogel was almost completely digested in 10 days under the action of proteinase K. However, the gel still retained more than 55% of its original weight in PBS solution without proteinase K after 14 days of incubation.

To investigate whether the introduction of TRSAW can confer positive charges to RADA16-I, we performed a zeta potential test (Figure 2G). In ultrapure water, the zeta potential of RADA16-TRSAW was 36.73 ± 1.50 mV; however, RADA16-I exhibited a nearly neutral charge of 2.71 ± 0.30 mV, indicating the positive charge of the RADA16-TRSAW peptide. This phenomenon was consistent with the theoretical isoelectric point as shown in Table S3. When mixed with RADA16-I, the zeta potential of the resulting mixture (TRSAWmax) still remained positive at 33.93 ± 1.19 mV. As expected, the formed TRSAWmax hydrogel also retained a stronger positive charge at 10.70 ± 0.56 mV compared with RADA16-I hydrogel (2.30 ± 0.11 mV). This indicates that more positive charge is exposed on the surface of the TRSAWmax hydrogel, which may help it adhere to more cells. Meanwhile, the zeta potential of the TRSAWmax hydrogel was reduced compared to its corresponding peptide solution. This phenomenon can be explained as follows. After hydrogel assembly, dense peptide stacking and β-sheet crosslinking partially encapsulate cationic functional groups within the three-dimensional network structure. The reduced exposure of positive surface charges ultimately causes a slight decrease in zeta potential compared with the original peptide solution.

### Cell adhesion capability of hBMSCs on self-assembled peptide hydrogels

The adhesive ability of the gel to cells was evaluated by DAPI nuclear staining (Figure 3A and B). To confirm the role of the positive charge of TRSAW in cell adhesion, we selected residue A (no charge) to replace residue R (positive charge) and designed a new polypeptide RADA16-TASAW (uncharged), to fabricate the TASAWmax (a mixture of RADA16-TASAW and RADA16-I solutions at a volume ratio of 1:1) hydrogel, which is electrically neutral (Table S3), as a control group. The cell adhesion assay results showed that the number of blue-stained cells in all groups increased over time. Clearly, the number of hBMSCs adhered to the TRSAWmax hydrogel was considerably higher than that on the RADA16-I hydrogel and TASAWmax hydrogel at 3 h, indicating that the electrostatic interaction between TRSAW and cells enhances cell adhesion to the gel. The overall trend shows that the adhesion rate is positively correlated with charge.

**Figure 3:**
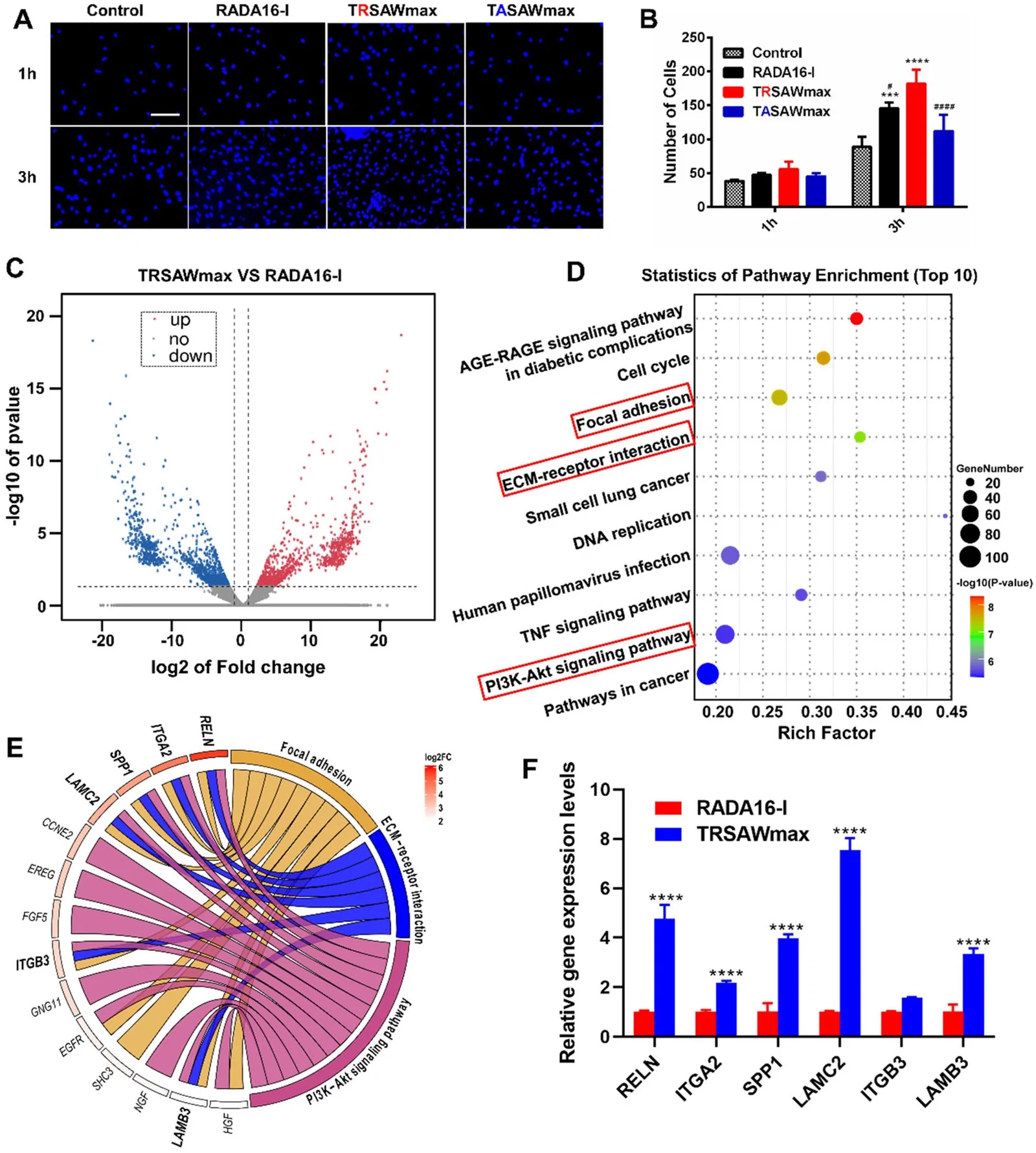
Effect of material surface charge on cell adhesion behavior. (**A**) The density of adherent cells incubated on the hydrogel for 1 or 3 h, determined by DAPI-staining. Scale bar = 200 μm. (**B**) The number of adherent cells was calculated using ImageJ. The data are presented as mean ± SD, with *n* = 6; ***P < 0.001 and ****P < 0.0001 vs. the control group, and ^#^*P* < 0.05 and ^####^*P* < 0.0001 vs. the TRSAWmax group. (**C**) Volcano plot of upregulated and downregulated genes in hBMSCs incubated with TRSAWmax hydrogel vs. RADA16-I hydrogel. (**D**) KEGG enrichment analysis of the transcriptome sequencing results. (**E**) The Circos map of the changed genes in adhesion-related KEGG enrichment pathways. (**F**) The relative gene expression levels of upregulated genes in adhesion-related GO enrichment pathways. The data are shown as mean ± SD, with *n* = 3, ****P < 0.0001 vs. the RADA16-I group.

To elucidate the profound effects of TRSAW addition on cell adhesion, we performed transcriptome sequencing analysis. Principal component analysis revealed a clear separation between the RADA16-I and TRSAWmax groups (Figure S8), demonstrating good biological reproducibility and differences between the groups. More than 2526 significantly different genes, including 1180 upregulated genes and 1346 downregulated genes, were identified in hBMSCs harvested from the TRSAWmax hydrogel compared with cells on the RADA16-I hydrogel (Figure 3C). These differentially expressed genes were further analyzed using the KEGG and GO databases. The top 10 KEGG-enriched pathways are shown in Figure 3D, and we found that cell adhesion-related items such as focal adhesion, ECM-receptor interaction, and the PI3K-Akt signaling pathway showed significant differences. In addition, four cell adhesion-related items (protein binding, integrin binding, cell adhesion, and focal adhesion) were enriched among the top 20 GO terms (Figure S9). These findings supported the view that the addition of TRSAW could effectively enhance cell adhesion. According to the gene–pathway interaction Circos map result (Figure 3E), we were surprised to find that the adhesion-related genes *RELN*, *ITGA2*, *SPP1*, *LAMC2*, *ITGB3* and *LAMB3* were involved in all three selected KEGG pathways, suggesting that these genes may play a crucial role in the adhesion initiated by TRSAWmax. Furthermore, the PCR results confirmed the upregulation of the adhesion-related genes (Figure 3F), which was consistent with the transcriptome sequencing results.

### Biocompatibility of self-assembled peptide hydrogels

The total number of viable hBMSCs was quantified using the CCK-8 assay (Figure 4A). As shown, hBMSCs proliferated in both peptide hydrogel scaffolds and in the control group. After 3 days of culture, no significant difference was observed between the control and the self-assembling peptide hydrogel scaffolds. After 5 days, the hydrogel scaffolds had significantly higher optical values than the control; however, the difference between RADA16-I and TRSAWmax was not clear after 9 days. As for cell viability (Figure 4B), there was no significant difference between RADA16-I and the functionalized self-assembling peptide hydrogel scaffolds (TRSAWmax) after 7 days. Routine blood test results and H&E staining *in vivo* also showed no statistically significant differences between groups, as shown in Figure 4C and D. These findings suggested that the peptide hydrogels had extremely low toxicity to hBMSCs and good biocompatibility.

**Figure 4:**
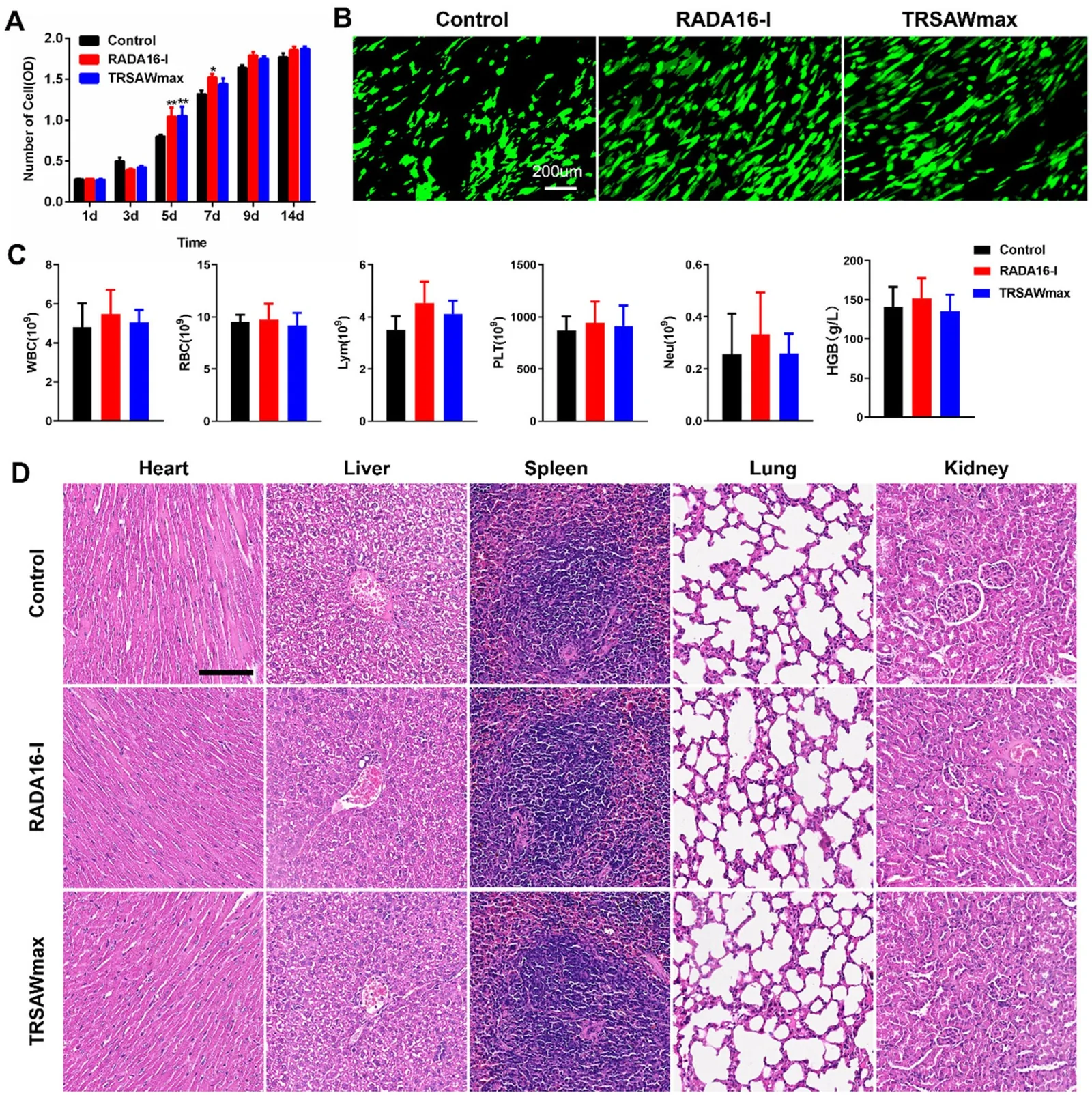
Preliminary toxicity and biocompatibility of hydrogel *in vitro* and *in vivo*. (**A**) OD values generated by the CCK-8 reagent for hBMSCs seeded in three groups after 1, 3, 5, 7, 9 or 14 days. *P < 0.05 and **P < 0.01 vs. the control group. (**B**) The survival of hBMSCs on different hydrogels was determined by CAM staining. Scale bar = 200 μm. (**C**) Results of routine blood analysis (WBC, white blood cell; RBC, red blood cell; Lym, lymphocyte; PLT, platelet; Neu, neutrophil; HGB, hemoglobin) and (**D**) H&E staining of the heart, liver, spleen, lung, and kidney in healthy mice after implantation of different hydrogel. Data are presented as the mean ± SD, with *n* = 3–5. Scale bar = 100 μm.

### Effects of hydrogels on the *in vitro* osteogenic differentiation of hBMSCs

The TRSAWmax hydrogels significantly promoted ALP expression compared with the RADA16-I and control groups (Figure 5A). Alizarin Red S (ARS) staining showed evidence of calcium deposition and nodule formation (Figure 5B). Although mineralized nodules were observed in all groups, more calcified nodules were found in hBMSCs stimulated by the TRSAWmax hydrogels on day 14. To further evaluate the effect of the hydrogel scaffold on osteogenic differentiation, gene and protein expression of osteogenic markers were examined. As listed in Figure 5C, mRNA expression levels of *ALP*, *OPN*, *OCN* and *Runx2* were dramatically higher in hBMSCs on TRSAWmax than on RADA16-I and the control on day 7. After 14 days, *OPN* expression in hBMSCs on TRSAWmax was lower than in the control; meanwhile, hBMSCs on TRSAWmax hydrogels expressed the highest level of *OCN*, a critical marker of late osteoblast differentiation. The protein expression levels of RUNX2, OCN and OPN followed the same pattern as the mRNA expression described previously (Figure 5D and E). These results indicated that TRSAWmax had better osteogenic performance than RADA16-I.

**Figure 5:**
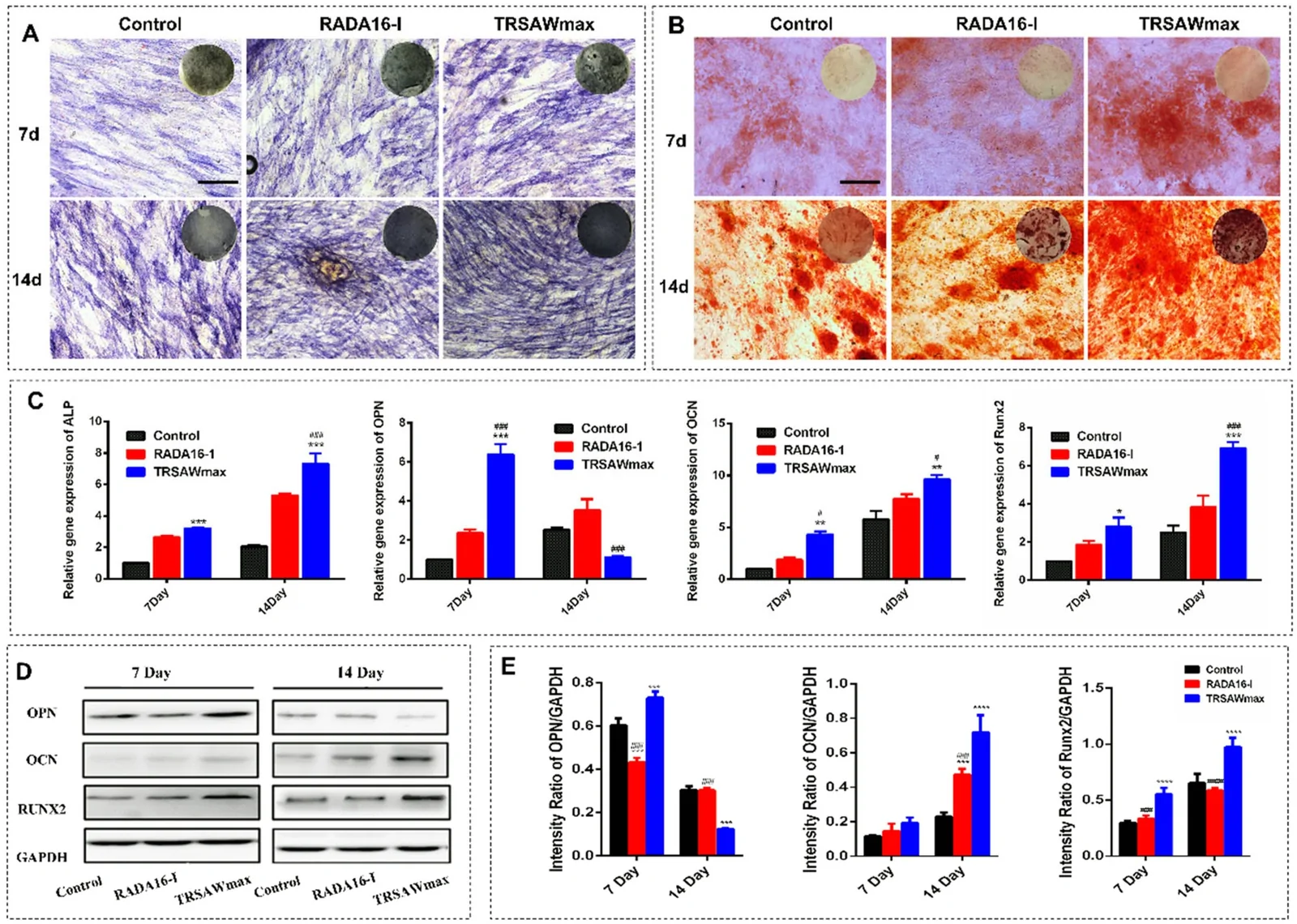
Effects of hydrogel scaffolds on the osteogenic differentiation of hBMSCs *in vitro*. (**A and B**) Osteogenic differentiation was assessed by ALP and Alizarin Red S staining on days 7 and 14. Scale bar = 200 μm. (**C**) The mRNA levels of Runx2, OPN, OCN and ALP after 7 and 14 days of culture. (**D and E**) Protein expression levels and quantitative results of RUNX2, OCN and OPN in hBMSCs seeded on different hydrogels for 7 and 14 days. GAPDH expression was used as an internal control. All data are expressed as mean ± SD and *n* = 3. *P < 0.05, **P < 0.01, ***P < 0.001 and ****P < 0.0001 vs. control group; ^#^*P* < 0.05, ^###^*P* < 0.001 and ^####^*P* < 0.0001 vs. the RADA16-I group.

### Effect of hydrogels on bone reconstruction *in vivo*

A murine bilateral femur-adjacent osteogenesis model was used to investigate the *in vivo* osteogenic capacity of the hydrogels, and Micro-CT analysis was performed to evaluate the new bone formation at 6 weeks postoperatively (Figure 6). The X-ray and 3D-reconstructed images showed more robust new bone formation in the TRSAWmax group than in the RADA16-I group and the control group (Figure 6A and B). Morphometric measurements were used to quantify new bone formation at the transplantation sites. The calculated parameters revealed that the BMD and the percentage of BV/TV, Tb. N and Tb. Th values in the TRSAWmax group were higher than those in the RADA16-I and control groups (Figure 6C).

**Figure 6:**
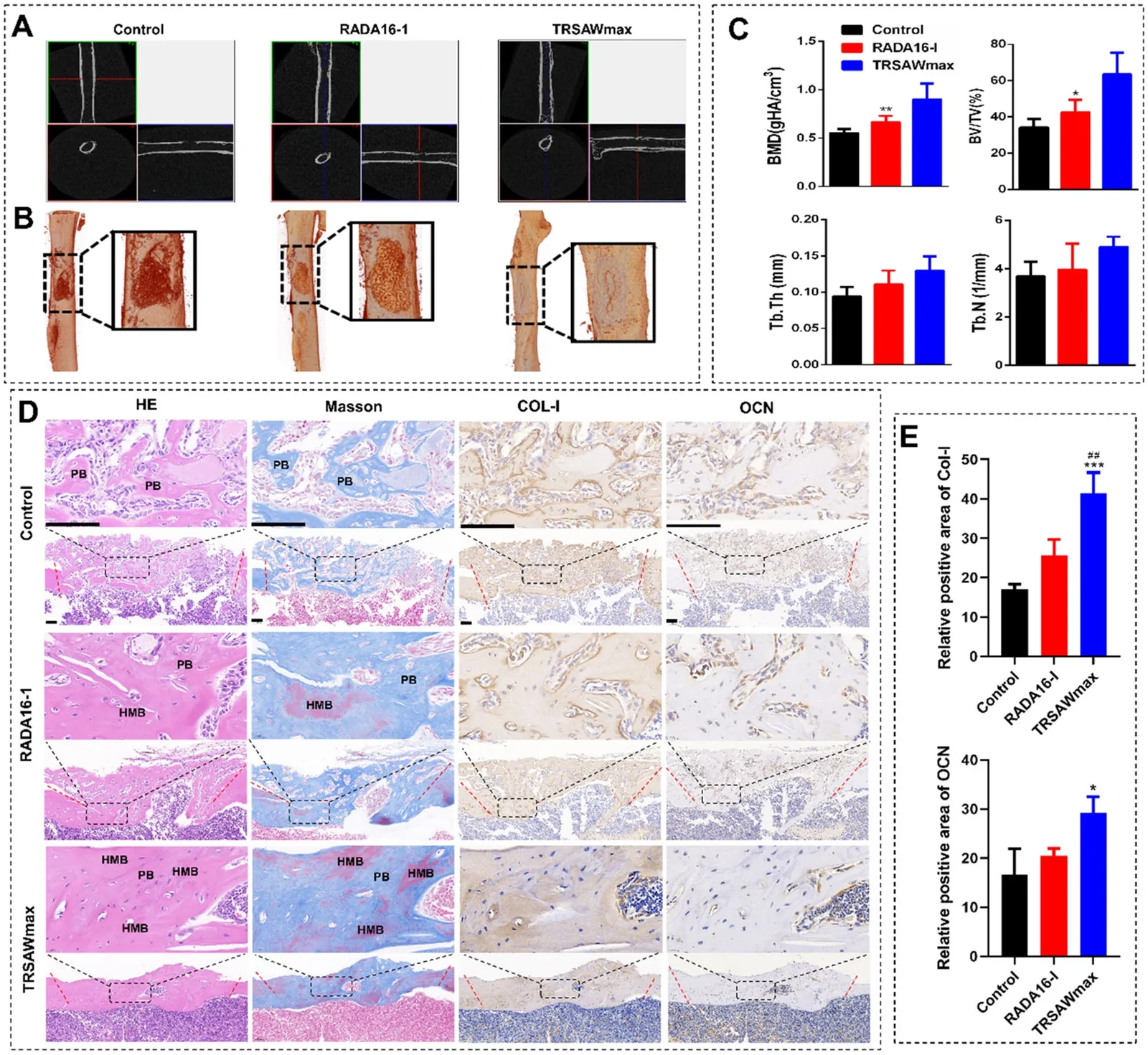
*In vivo* bone regeneration effects of the hydrogel scaffolds. (**A and B**) Representative X-ray and 3D-images reconstructed by micro-CT. (**C**) Morphometric analysis of BMD, BV/TV, Tb. Th and Tb. N. (**D**) Histological analysis including H&E staining, Masson staining and immunohistochemical staining for COL-I and OCN of decalcified bone slices collected after 6 weeks of implantation. PB: primary bone (blue) and HMB: highly mineralized bone (red). Scale bar = 100 μm. (**E**) Quantitative analysis of the positive areas in immunohistochemical staining. All data are expressed as mean ± SD, with *n* = 3. *P < 0.05, **P < 0.01 and ***P < 0.001 vs. the control group; ^##^*P* < 0.01 vs. the RADA16-I group.

H&E and Masson staining revealed that the defect regions in the control group were covered with a small amount of primary bone (PB) and some soft tissue (Figure 6D). In contrast, the defects in the RADA16-I and TRSAWmax hydrogel groups were bridged by not only a large amount of PB but also highly mineralized bone (HMB). Additionally, more HMB was observed in the TRSAWmax hydrogel-treated groups, indicating that the relative bone healing ratio was highest in the TRSAWmax hydrogel group. The immunohistochemical (IHC) staining of representative osteoblast markers (COL-I and OCN) was analyzed to assess the quality of newly formed bone tissue (Figure 6D). The IHC images and quantification results (Figure 6E) showed that COL-I and OCN were highly expressed in TRSAWmax hydrogel-treated defect regions, indicating greater collagen deposition and mineralization of the bone extracellular matrix, which further confirmed that the TRSAWmax hydrogel accelerated new bone maturation.

### The molecular mechanism by which the TRSAWmax hydrogel repairs bone defects

As shown in Figure 7A and C, the TRSAWmax hydrogel significantly increased the levels of phosphorylated Src and Erk1/2, as well as the osteogenic markers COL-I and the bone formation-related protein VEGF, compared with the control and RADA16-I groups. Importantly, the expression of the osteogenesis-related protein in hBMSCs on the TRSAWmax hydrogel was reversed in the presence of a Src inhibitor or an Erk1/2 inhibitor, which effectively suppressed the phosphorylation of Src and Erk1/2 (Figure 7B and D). Additionally, after applying the Src inhibitor, the protein expression levels of Erk and its phosphorylation were greatly inhibited, whereas the levels of Src and its phosphorylation were much less affected, demonstrating that Src was upstream of Erk (Figure 7B and D). Furthermore, to further illustrate the influence of the Src-Erk pathway on promoting the expression of osteogenic differentiation proteins and mineralization in hBMSCs, we performed ALP staining and ARS staining (Figure 7E). Along with the Src inhibitor or Erk1/2 inhibitor, a significant reduction in ALP staining as well as a decrease in stained calcium nodules and the stained area by alizarin red, was observed compared with the inhibitor-deficient group (TRSAWmax).

**Figure 7:**
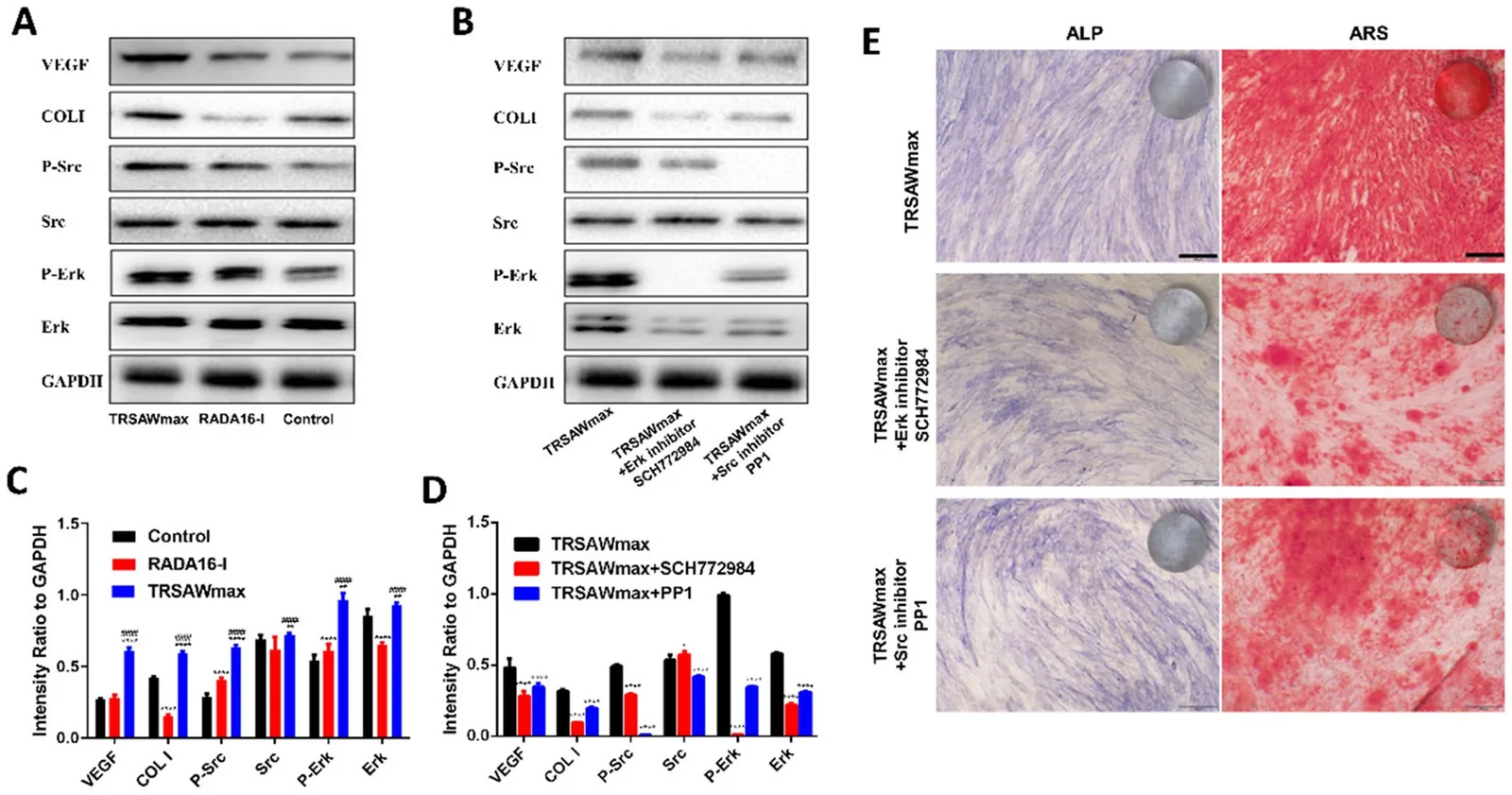
Study of the molecular mechanism by which the TRSAWmax hydrogel repairs bone defects. (**A and C**) Protein expression levels and quantitative results of VEGF, COL-I, P-Src, Src, P-Erk and Erk for different hydrogels. (**B and D**) Protein expression levels and quantitative results of VEGF, COL-I, P-Src, Src, P-Erk and Erk for the TRSAWmax hydrogel with or without the Src inhibitor PP1 or the Erk inhibitor SCH772984. All data are expressed as the mean ± SD, with *n* = 3. *P < 0.05, **P < 0.01 and ****P < 0.0001 vs. control group; ^####^*P* < 0.0001 vs. the RADA16-I group. (**E**) ALP and Alizarin Red S staining of hBMSCs treated with PP1 or SCH772984 on the TRSAWmax hydrogel. Scale bar = 200 μm.

## Discussion

The widespread use of modern transportation and the trend toward an aging society have gradually increased the incidence of orthopedic disorders caused by trauma and aging, creating a huge social and economic burden. Autografts and allografts are widely used clinically for bone defects. However, several limitations restrict the use of bone grafts, including tissue source, donor site bone defects, immune reactions and infection [32, 33]. Advances in bioactive materials have provided new treatments for orthopedic disorders, and various types of scaffolds are clinically applicable for enhancing the treatment of large and critically sized bone defects [10–12, 34]. Self-assembled peptides offer excellent biocompatibility, adjustable mechanical stability, injectability, responsiveness to triggers and the capacity to carry cells and active pharmaceutical agents for tissue regeneration. Therefore, they have been widely used as tissue engineering scaffolds [35, 36]. RADA16-I peptide can self-assemble into a network microstructure that provides a favorable 3D microenvironment for various tissue engineering applications [16, 37]. Moreover, RADA16-I can mimic the architecture of natural ECM and enhance cell viability, proliferation, and differentiation when combined with biologically active molecules. Compared with traditional modification techniques, such as conventional encapsulation and physical attachment, directly adding biofunctional short peptides to RADA16-I can achieve long-term retention of growth factors owing to the outstanding interactions between biomolecules and cells [17, 38]. PTH is an 84-amino-acid polypeptide that controls the balance of calcium reabsorption and calcium release [39, 40]. Several bioactive epitopes have been identified in PTH. It is worth noting that among these epitopes, TRSAW (PTHrP (107–111)) exhibits excellent osteogenic properties *in vitro* and the ability to stimulate bone accrual *in vivo* [25, 29, 31], as well as a net positive charge. We demonstrate that incorporating TRSAW into RADA16-I self-assembling hydrogel scaffolds could enhance their adhesive and osteogenic properties for implantation applications.

Firstly, we innovatively grafted the short peptide TRSAW onto the self-assembling backbone of RADA16-I via solid-phase synthesis in this study. The successful preparation of the composite RADA16-TRSAW was confirmed through HPLC and MS analyses. The CD spectra and AFM observation results showed that conjugating the small functional peptide TRSAW to the C-terminus of RADA16-I destabilized the structure of the RADA16-TRSAW peptide and weakened the formation of an interlinked network, making it difficult to form a durable hydrogel. These results may be attributed to the random coil structure of the functionalized peptide RADA16-TRSAW and were consistent with previous studies [41, 42]. It has been reported that a pure modified RADA16-I solution could not form a stable hydrogel owing to the difficulty of forming an interlinked network, whereas incorporating RADA16-I at a proportion higher than 50% solved this problem, and the stability increased as the RADA16-I content increased [23]. In our study, when mixed with RADA16-I at a 1:1 volume ratio, the resulting TRSAWmax recovered a high β-sheet content and nano-fiber crosslinking points similar to those of RADA16-I, and TRSAWmax peptide solutions were able to spontaneously assemble into a durable hydrogel induced by salt ions (Figure 2A–C). The release behavior of polypeptides in PBS demonstrated the stability of the gel. The burst release observed within 1 h in both the RADA16-TRSAW and TRSAWmax groups may be attributed to the diffusion of free peptides that had not been assembled into the scaffold; the slow release within 10 h was due to the dissolution of loosely linked peptides; and the stable state within 72 h indicated that the morphology remained stable after self-assembly into a hydrogel. A comprehensive comparison of the characterization of the RADA16-TRSAW and TRSAWmax groups showed that RADA16-TRSAW was less stable than TRSAWmax; therefore, TRSAWmax was selected for the following experiments because stable 3D hydrogel scaffolds allowed adequate preparation for cell culture.

Studies have shown that charge affects cell behavior [43, 44], including adhesion, spreading, proliferation, migration, polarization, differentiation, apoptosis, necrosis and signal transduction [45]. First, Trejo *et al.* [30] reported that PTHrP 107–111 (TRSAW) contained positive charges and TRSAW-loaded bioceramics significantly improved local bone induction, but they did not reveal the effect of charge on cells. Therefore, in this study, we hypothesize that the obtained polypeptide RADA16-TRSAW may exert its effect by promoting cell adhesion through the interaction between positive and negative charges. To confirm this, we established an uncharged control group TASAW by replacing the positively charged arginine (R) in TRSAW with uncharged alanine (A) to mask the charge. The charge level was evaluated using a zeta potentiometer (Figure 2G). Using hBMSCs to assess adhesion efficiency, the results showed that the TRSAWmax group had greater cell adhesion than the other groups, with a statistically significant difference, whereas the TASAWmax group showed no significant difference in adhesion efficiency compared with RADA16-I. Furthermore, transcriptome sequencing analysis found that TRSAWmax could upregulate genes and activate pathways related to adhesion (Figure 3). The results that positive charge of functional short peptides can enhance cell adhesion and provide a foundation for osteogenic differentiation have been confirmed by this study and previous studies [46–48]. However, the specific membrane receptors through which the positively charged surface of TRSAWmax triggers intracellular adhesion-related signaling pathways remain unclear in this study, representing a key scientific issue to be addressed in future research.

Multiple aspects of the synergistic biological effects of the hydrogel scaffolds were evaluated. As a bone repair scaffold, its biodegradation and toxicity behaviors are of crucial importance. Our hydrogel system demonstrated excellent stability in PBS and showed a tendency to gradually degrade under the action of proteinase K. Its moderate enzymatic degradation rate may align well with the speed of new bone formation, enabling gradual replacement of the scaffold by newborn bone tissue in the later stage [49]. The artificial microenvironment may cause cell death due to the toxicity of the biomaterial and improper handling. However, the TRSAWmax gel was non-toxic to cells, and the cells showed healthy growth (Figure 4). Even after the gel is implanted into the body, it does not trigger inflammatory responses in major organs such as the heart, liver, spleen, lungs and kidneys. There are also no clear inflammatory indicators in the blood. This indicates that the material has good biocompatibility. Self-assembling peptide hydrogels, as an artificial extracellular matrix, can promote cell infiltration and adhesion and have been widely used in regenerative medicine, including nerve regeneration, spinal cord injury repair and bone tissue engineering [16, 17]. RADA16-I, a clinically approved self-assembling peptide (PuraMatrix™), has been shown to support mature bone formation in calvarial bone defects in mice [50]. To further enhance bone formation, active proteins such as bFGF and BMP-2 were incorporated into the RADA16 hydrogel, successfully accelerating bone regeneration [51, 52]. He *et al.* [53] demonstrated that an RADA16 hydrogel functionalized with the cell-adhesive RGD motif and loaded with bFGF can effectively promote osteoblast spreading and bone healing in an SD rat femoral condyle defect model. Our experimental results confirmed that modifying the TRSAW short peptide in RADA16 increases the number of cells adhering to the gel surface and enhances osteogenic differentiation ability *in vitro* and *in vivo*. A potential explanation for this phenomenon is the presence of the TRSAW sequences, which contain positive charges that enable cells to adhere through electrostatic interactions and promote osteogenic differentiation [25, 30, 54]. Compared with previous studies, our cationic peptide-modified RADA16 hydrogel exhibits the dual functions of adhesion and osteogenesis, along with ease of preparation and simplicity of composition.

Previous reports have suggested that TRSAW-induced Src kinases mediate VEGFR2 transactivation to modulate osteoblastic function [25, 55]. Here, this study explored the mechanism by which TRSAWmax promotes the osteogenic differentiation of hMSCs. VEGF and COL-Ι were chosen as indicators because they play important roles in osteogenic differentiation, and COL-Ι is associated with cell adhesion and growth. The highest expression of VEGF and COL-I corresponded to the highest activation of Src and Erk1/2 phosphorylation in the TRSAWmax group, but upon inhibition of Src or Erk1/2 kinase, the expression of the early osteogenic proteins VEGF and COL-Ι was lower than in the uninhibited group (Figure 7). Importantly, when Src was inhibited, the expression of Erk and its phosphorylation were markedly decreased, whereas the effect on Src was small when Erk was inhibited. Those findings verified that Src was upstream of Erk and that the Src-Erk signaling pathway played an important role in modulating osteogenic function by TRSAWmax.

## Conclusion

In conclusion, the TRSAWmax hydrogel fabricated by RADA16-I and RADA16-TRSAW can promote the adhesion of cells on its surface with active positive charged groups, and may be in favor of retaining osteoblast-related cells in the defect site to accelerate bone formation with excellent osteogenic ability. The TRSAWmax hydrogel exhibits good osteogenic differentiation and bone repair ability *in vitro* and *in vivo*, which is probably related to the phosphorylation and activation of the Src-Erk signaling pathway. The TRSAWmax hydrogel is a promising treatment option for bone defect repair, especially for non‑load‑bearing lacunar bone defects and alveolar bone repair. Given its excellent cell adhesion performance, TRSAWmax hydrogel represents a promising candidate for selective cell retention technology, used as a scaffold for enriching bone marrow cells to enhance bone repair outcomes. Moreover, in light of its insufficient mechanical strength, it can be composited with other mechanically robust materials to broaden its clinical applicability.

Nevertheless, several limitations remain to be addressed. First, the specific molecular mechanism between the charge stimulation and the cell is not explored in this study, and whether it may cause a concatenation of transmembrane charge reaction is also unknown, and still needs to be studied. Second, the pathways underlying osteogenesis are pivotal to bone regeneration. Further studies are required to clarify the more detailed pathways underlying the promoted osteogenic differentiation *in vitro*. Additionally, self-assembled gels alone are not fully suitable for treating large bone defects owing to their mechanical limitations. Repairing bone defects with self-assembled gels is an emerging challenge that may be solved through the development of novel complex biomaterials. In any case, the therapeutic potential of self-assembling hydrogels is evident, and in-depth research on RADA16-I peptides is ongoing and expected to provide further insights into biomaterial development and bone tissue engineering applications.

## Supplementary Material

rbag162_Supplementary_Data

## Data Availability

Data will be made available on request.
