# Supplementary material for: Cation-surface RADA16-based self-assembling peptide hydrogel with high adhesion and osteogenesis promoting bone regeneration
Source: Regen Biomater. 2026 Aug 4;13:rbag162. doi: 10.1093/rb/rbag162 (PMC13633681; doi:10.1093/rb/rbag162)
Supplement: Supplementary file 1 [file rbag162_supplementary_data.docx]

**SUPPLEMENTARY INFORMATION**

**Cation-surface RADA16-based self-assembling peptide hydrogel with high adhesion and osteogenesis promoting bone regeneration**

Jiangling Zhou^1,a,b,c^, Jing Gou^1,a,c,d^, Qiandong Yang^1,a,b,c^, Sihao He^a,b,c^, Juan Cai^a,b,c^, Qiankun Yang^a,b,c^, Bo Yu^a,b,c^, Qijie Dai^a,b,c^, Jiulin Tan^a,b,c^, Yingtao Gui^a,b,c^, Yuan Guo^e^, JunchaoXing^a,b,c^, Tianyong Hou^a,b,c^

*a Department of Orthopaedics, Southwest Hospital, Army Medical University (Third Military Medical University), Chongqing 400038, China*

*b Chongqing Engineering Research Center for Advanced Material Design and Intelligent Manufacturing in Bone Repair and Reconstruction, Chongqing 400038, China*

*c National & Regional United Engineering Lab of Tissue Engineering, Department of Orthopaedics, Southwest Hospital, Army Medical University (Third Military Medical University), Chongqing 400038, China*

*d Sichuan College of Traditional Chinese Medicine，Chengdu 610065，China*

*e Department of Orthopaedics, 958th Hospital of Chinese People’s Liberation Army (Third Military Medical University), Chongqing 400038, China*

Corresponding author：Tianyong Hou, PhD, Associate Prof.

Department of Orthopaedics,

Southwest Hospital,

Army Medical University (Third Military Medical University), Chongqing 400038, China

ORCID: 0000-0002-7685-2088

1 These authors contributed equally to this work.

29 GaotanyanStreet, Shapingba District, Chongqing 400038, (China)

Tel. +8615023253626, E-Mail [tianyonghou@tmmu.edu.cn](mailto:tianyonghou@tmmu.edu.cn).

**Figure S1** The high-performance liquid chromatography (HPLC) peaks of RADA16-I and RADA16-TRSAW peptide.

**Figure S2** The mass spectrum (MS) of RADA16-I and RADA16-TRSAW peptide.

**Figure S3** Macroscopic appearance images of RADA16-TRSAW solution before (left) and after (right) trigged by PBS.

**Figure S4** Photos of the double-syringe system and the injectability of the TRSAWmax hydrogel.

**Figure S5** Cryo-scanning electron microscope (Cryo-SEM) micrograph of RADA16-I and TRSAWmax solution.

**Figure S6** Rheological measurements of RADA16-I and TRSAWmax hydrogel over a strain of 0.1−50%.

**Figure S7** Degradation curves of TRSAWmax hydrgels in the absence or presence of proteinase K.

**Figure S8** The principle component analysis（PCA）of the transcriptome sequencing results.

**Figure S9** The GO enrichment analysis of the transcriptome sequencing results.

**Table S1.** HPLC peak results of RADA16-I and RADA16-TRSAW peptide.

|  | **Peak** | **Ret.Time** | **Area** | **Conc. (%)** |
| --- | --- | --- | --- | --- |
| RADA16-I | 1 | 11.178 | 2107607 | 93.03 |
|  | 2 | 11.453 | 157806 | 6.966 |
| RADA16-TRSAW | 1 | 10.073 | 87161 | 0.6577 |
|  | 2 | 10.323 | 12358810 | 93.26 |
|  | 3 | 10.587 | 806153 | 6.083 |

**Table S2.** The quantification of secondary structures for RADA16-I, RADA16-TRSAW and the composite TRSAWmax.

| **Sample** | **α-helix** | **β-sheet** | **β-turn** | **Random** |
| --- | --- | --- | --- | --- |
| RADA16-I | 9.8% | 58.8% | 7.0% | 24.5% |
| RADA16-TRSAW | 9.2% | 25.9% | 15.3% | 49.6% |
| TRSAWmax | 12.1% | 40.2% | 12.4% | 35.3% |

**Table S3.** Physicochemical properties for different peptides solution and hydrogels.

| **Name** | **Isoelectric point** | **Total Number of negatively charged residues （Asp+Glu）** | **Total Number of positively charged residues （Arg+Lys）** | **Net charge** | **Zeta Potential（mV）** |
| --- | --- | --- | --- | --- | --- |
| RADA16-I | 6.10 | 4 | 4 | 0 | 2.71±0.30 |
| RADA16-TRSAW | 9.40 | 4 | 5 | +1 | 36.73±1.50 |
| RADA16-TASAW | 6.10 | 4 | 4 | 0 | -17.47±0.29 |
| TRSAWmax |  | 8 | 9 | +1 | 33.93±1.19 |
| TASAWmax |  | 8 | 8 | 0 | -5.58±0.32 |
| RADA16-I hydrogel |  | 4 | 4 | 0 | 2.30±0.11 |
| TRSAWmax hydrogel |  | 8 | 9 | +1 | 10.7±0.56 |
| TASAWmax hydrogel |  | 8 | 8 | 0 | -7.73±0.18 |

**Table S4.** Primer sequences used for qRT-PCR analysis.

| **Genes** | **Species** | **Forwardprimer (5’-3’)** | **Reverseprimer (5’-3’)** |
| --- | --- | --- | --- |
| *GAPDH* | Human | CAGGAGGCATTGCTGATGAT | GAAGGCTGGGGCTCATTT |
| *ALP* | Human | GACCTCCTCGGAAGACACTC | TGAAGGGCTTCTTGTCTGTG |
| *OCN* | Human | AGCAAAGGTGCAGCCTTTGT | GCGCCTGGGTCTCTTCACT |
| *OPN* | Human | TCGCAGACCTGACATCCAGTACC | AATCTGGACTGCTTGTGGCTGTG |
| *RUNX2* | Human | GCATGGAATCTCTGGTGGAAG | TGAGTGGTGCCGTACTGGTA |
| *RELN* | Human | CGTCCTAGTAAGCACTCGCA | TCGCCTAAGTGACCTTCGTC |
| *ITGA2* | Human | ATTCTCCCTGCCGGTTGATG | CAGGGTAGCCTACATCGCAG |
| *SPP1* | Human | GCAGCTTTACAACAAATACCCAGA | ACTTGGAAGGGTCTGTGGGG |
| *LAMC2* | Human | TACTGACTGCTGCTGCCTTG | ACTGTCCCACTCCAAACCTG |
| *ITGB3* | Human | TGTACCACGCGTACTGACAC | CTGCCACATTCACACTTGCC |
| *LAMB3* | Human | CTTTGACCCCGCTGTGTTTG | CTGACACCGCTCACAGTTCT |
